# Ultrafast Dynamics of Extraordinary Optical Transmission through Two-Slit Plasmonic Antenna

**DOI:** 10.3390/nano13162284

**Published:** 2023-08-09

**Authors:** Guangqing Du, Fangrui Yu, Yu Lu, Lin Kai, Caiyi Chen, Qing Yang, Xun Hou, Feng Chen

**Affiliations:** 1 State Key Laboratory for Manufacturing System Engineering and Shaanxi Key Laboratory of Photonics Technology for Information, School of Electronic Science and Engineering, Xi’an Jiaotong University, Xi’an 710049, China; guangqingdu@mail.xjtu.edu.cn (G.D.); yfr1999@stu.xjtu.edu.cn (F.Y.); luyu90@xjtu.edu.cn (Y.L.); 3120105005@stu.xjtu.edu.cn (L.K.); moraynia@stu.xjtu.edu.cn (C.C.); houxun@mail.xjtu.edu.cn (X.H.); 2 School of Mechanical Engineering, Xi’an Jiaotong University, Xi’an 710049, China; yangqing@mail.xjtu.edu.cn

**Keywords:** ultrafast dynamics, extraordinary optical transmission, phase correlation, femtosecond laser

## Abstract

We have theoretically investigated the spatial-temporal dynamics of extraordinary optical transmission (EOT) through a two-slit plasmonic antenna under femtosecond laser dual-beam irradiation. The dynamic interference of the crossed femtosecond laser dual-beam with the transiently excited surface plasmon polariton waves are proposed to characterize the particular spatial-temporal evolutions of EOT. It is revealed that the dynamic EOT can be flexibly switched with tunable symmetry through the respective slit of a two-slit plasmonic antenna by manipulating the phase correlation of the crossed femtosecond laser dual-beam. This is explained as tunable interference dynamics by phase control of surface plasmon polariton waves, allowing the dynamic modulation of EOT at optimized oblique incidences of dual-beams. Furthermore, we have obtained the unobserved traits of symmetry-broken transient spectra of EOT from the respective up- and down-slit of the antenna under crossed femtosecond laser dual-beam irradiation. This study can provide fundamental insights into the ultrafast dynamics of EOT in two-slit plasmonic antennas, which can be helpful to advance a wide range of applications, such as ultrafast plasmonic switch, ultrahigh resolution imaging, the transient amplification of non-linear effects, etc.

## 1. Introduction

Extraordinary optical transmission (EOT) through a variety of subwavelength nanostructures in metal films has attracted great interest for both scientific and practical purposes [1,2,3,4,5,6]. EOT refers to enhanced optical transmission with improved efficiencies exceeding unity at a certain wavelength, which are orders of magnitude higher than the transmission predicted by classical diffraction theory. As the simplest geometry, the subwavelength slits or holes hold great promise for exploring the fundamental physics of EOT and enabling potential applications in areas such as highly sensitive bio-sensors, ultrafast light switch, non-linear enhanced imaging, etc. [7,8,9,10].

Physically, surface plasmon and cavity mode resonances, known as Fabry–Perot (FP) resonance, play a dominant role in the formation of EOT. Most previous research focuses on investigations of the static-state modes of EOT in terms of geometry, size, and the composition of nanostructured materials, as well as the wavelength and polarization of the incident light [11,12,13,14,15,16]. However, as for the dynamic behavior of EOT, it is currently an ongoing topic for exploring the potential capability to actively and flexibly manipulate the EOT in spatial-temporal regimes [17]. Recently, the femtosecond laser has discovered great potential for manipulating the dynamic properties of the surface plasmon resonance of nano-geometry [18,19,20,21,22]. Müller et al. theoretically investigated the dynamics on a metallic transmission grating with femtosecond laser illumination and demonstrated the special importance of surface plasmon polariton (SPP) waves for controlling the spectral properties of EOT [23]. Joly et al. revealed that tuning the pulse-to-pulse separation can be qualified to effectively control the SPP dynamics with respect to femtosecond laser dual-pulse excitation [24]. The spatial-temporal dynamics of the photo-excitation of fused silica by means of femtosecond laser dual-pulse were thoroughly investigated by Pan et al. [25]. Based on this, it is strongly hypothesized that the SPP dynamics tend to be able to be deliberately manipulated by dual-beam femtosecond laser irradiation to support EOT. Unfortunately, due to the complex dynamic mechanisms controlling EOT in the spatial-temporal regimes, it still raises a serious limitation for understanding the transient properties of EOT. In particular, it is currently a challenge to explore the ultrafast dynamics of EOT in terms of the pulse-to-pulse correlation of femtosecond laser dual-beam excitation. However, to the best of our knowledge, the ultrafast dynamics of EOT under light excitation by femtosecond laser dual-beam is not yet well explored in terms of pulse-to-pulse phase correlation.

This paper reports the theoretical work on the ultrafast dynamics of EOT using a two-slit plasmonic antenna milled on a Au film on top of a silica substrate. It is proposed that the dynamic interferences between the femtosecond laser dual-beam and the transiently excited SPP waves are functioned to regulate particular aspects of the spatial-temporal characteristics of EOT from a two-slit plasmonic antenna. It is observed that the transient EOT by the two-slit plasmonic antenna exhibits symmetry-tunable properties, which can be flexibly switched by manipulating the phase correlation of the crossed femtosecond laser dual-beam. We further explore the transient spectra of EOT with symmetry-tunable properties from the respective up- and down-slit of the antenna under crossed femtosecond laser dual-beam irradiation.

## 2. Modelling and Methods

**Figure 1:**
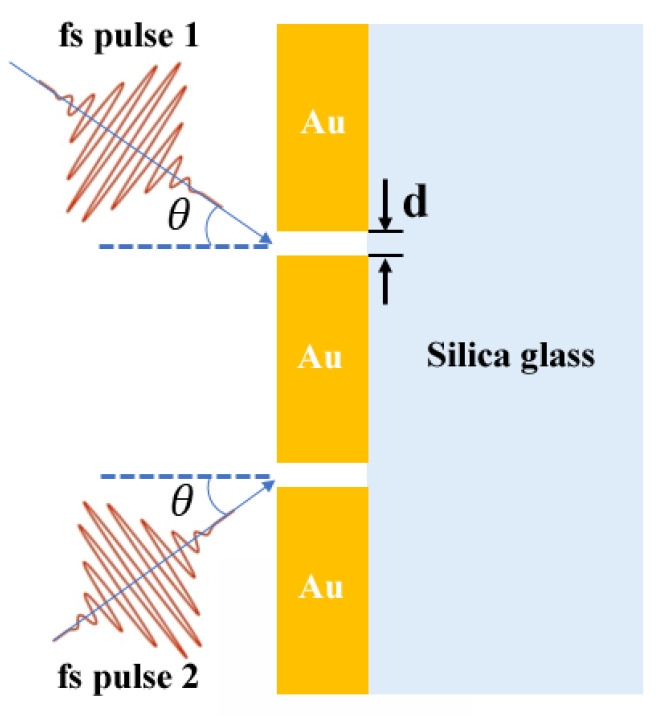
A schematic of the two-slit plasmonic antenna milled in a Au film on top of silica substrate excited by a crossed femtosecond laser dual-beam.

A schematic of the two-slit plasmonic antenna milled in a Au film on top of a silica substrate excited by a crossed femtosecond laser dual-beam is shown in Figure 1. Here, we applied 2-D geometry to save the computation load, but keep the fundamental characteristics of the dynamic EOT of the two-slit plasmonic antenna. Two beams of a TM-polarized femtosecond (fs) laser pulse with a respective angle of incidence of θ occur simultaneously on the geometry of a two-slit plasmonic antenna. The slit width w of the two-slit plasmonic antenna is set to 250 nm, about a quarter of the central wavelength of 1.03 μm for the incident femtosecond laser. The distance between the two slits is set to 4 μm. The thickness of the Au film is fixed at 1 μm. In this work, however, the interesting element lies in the time dynamic behaviors of EOT. As a result, we adopted the optimized geometry (metal film thickness 1 μm, slit width 250 nm, slit distance 4 μm) for outputting the electric field at a designed infrared wavelength for focusing the topic of the ultrafast dynamics of EOT through a two-slit plasmonic antenna. The phase difference ∆φ and incident angle θ of the crossed femtosecond laser dual-beam are used as variables to study the effects of the particular phase correlation of the crossed femtosecond laser dual-beam on the spatial-temporal characteristics of EOT.

In this work, the transient surface plasmon polariton waves excited by a femtosecond laser dual-beam that simultaneously couple with the dual-beam are dynamically modeled based on the transient wave equation by applying the Drude Lorentz dispersive media model to the two-slit plasmon antenna. The full transient wave equation can be typically described in terms of the magnetic vector potential $\vec{A}$ as follows:

(1) $$\nabla \times \mu_{r}^{-1}\left(\nabla \times \vec{A}\right)+\mu_{0}\sigma \frac{\partial \vec{A}}{\partial t}-\mu_{0}\frac{\partial \vec{D}}{\partial t}=0\;$$

where $\mu_{r}$ is the relative permeability taken as 1 for the materials here, $\mu_{0}$ is the permeability in vacuum, and σ is the electric conductivity. The electric displacement vector $\vec{D}\;$ is defined by:

(2) $$\vec{D}=\varepsilon_{0}\varepsilon_{\infty }\vec{E}\;+\vec{\;P}\;$$

Here, $\vec{E}$ denotes the electric field vector. The frequency-dependent dielectric function described by the Drude Lorentz term [26,27] can be written as:

(3) $$\varepsilon_{r}=\varepsilon_{\infty }-\frac{\omega_{p}^{2}}{\omega^{2}-j\Gamma_{D}\;\omega }-\frac{\Delta \epsilon \Omega_{L}^{2}}{(\omega^{2}-\Omega_{L}^{2})-j\Gamma_{L}\;\omega }$$

where $\;\varepsilon_{\infty }\;$ is the high-frequency dielectric constant, $\omega_{p}$ is the plasma frequency, ω is the central frequency of the incident femtosecond laser dual-beam, $\Gamma_{D}$ is a damping coefficient, Δϵ is the weighting factor, and $\Omega_{L}$ and $\Gamma_{L}$ explain the oscillator strength and the spectral width of the Lorentz oscillator, respectively. The values of relevant parameters for this simulation are listed as follows:$\;\varepsilon_{\infty }=5.9673,\;\omega_{p}/2\pi =2113.6\times 10^{12}$ Hz,${\;\Gamma }_{D}/2\pi =15.92\times 10^{12}$ Hz,${\;\Omega }_{L}/2\pi =650.07\times 10^{12}\;\mathrm{Hz}$, $\Gamma_{L}/2\pi \;$ = 104.86 $\times 10^{12}\;\mathrm{Hz}$, Δϵ=1.09.

Here, $\vec{P}$ is the polarization field which can be described by an ordinary differential equation, written as:

(4) $$\left(\frac{\partial^{2}}{\partial t^{2}}+\Gamma_{D}\frac{\partial }{\partial t}+\omega^{2}\right)\vec{P}=\varepsilon_{0}f\omega_{p}^{2}\vec{E}$$

where *f* is the oscillator strength normalized as 1.

To simplify the simulations, the electric field of the crossed femtosecond laser dual-beam is assumed to be a plane wave with a Gaussian temporal shape defined by:

(5) $$E=\sum_{i=1}^{2}E_{0i}\exp[jk\left(x\cos\theta +y\sin\theta \right)+j\varphi_{i}]\;exp\left[-{\left(\frac{t-t_{0}}{t_{p}}\right)}^{2}\right]\;\;$$

Here, $E_{0i}$ is the electric field amplitude of the Gaussian wavelet for the ith pulse of the dual-beam. *k* is the modulus of wavevector, written by 2π/λ, with λ denoting the wavelength of incident laser. θ is the respective angle of incidence of the crossed femtosecond laser dual-beam, $\varphi_{i}$ is the phase for the respective pulse of the dual-beam, $t_{p}$ is the pulse duration which is assumed to be identical to the respective pulse of the dual-beam. We solve the dynamically coupled equations of (1)–(5) using the RF module of commercial COMSOL Multiphysics. Firstly, we build a 2D geometry of the two-slit plasmonic antenna. Meanwhile, we define the index of refraction on the different regions of the antenna geometry. In particular, the index of refraction of Au is calculated by $\sqrt{\varepsilon_{r}}$, in which $\varepsilon_{r}$ is the frequency-dependent dielectric function described by the Drude Lorentz term described in Equation (3). Secondly, the full transient wave equation as seen in Equation (1) is totally applied on the regions of air, silica glass, and the two-slit plasmonic antenna, respectively. In addition, Equation (4) for solving the polarization field $\vec{P}$ is defined in the region of two slits of Au film, which play a key role in forming the surface plasmon polariton waves for the support of EOT. Thirdly, we define the initial and boundary conditions. The initial conditions are taken as $\vec{A\;}$= 0 and $\partial \vec{A}/t=0$. The laser source is taken as the input boundary condition on the front surface of the calculation geometry. The interior boundaries between the Au film slit and surrounding medium are taken as the perfect electric/magnetic conductor, generally written as $\vec{n}\times \vec{E}$ = 0, $\vec{n}\times \vec{H}$ = 0, or $\vec{n}\times ({\vec{E}}_{1}-{\vec{E}}_{2})=0$, $\vec{n}\times ({\vec{H}}_{1}-{\vec{H}}_{2})=0$. Here, $\vec{n}$ denotes the normal direction at the interface, the lower markings 1 and 2 represent the Au film and the surrounding medium, respectively. On the bottom of the silica substrate, however, we adopt a scattering boundary condition with no incident wave, described by $\mu_{0}\vec{n}\times \vec{H}+\mu_{0}/Z_{c}\vec{n}\times (\vec{E}\times \vec{n})$=$\;\;\gamma /\mu_{r}\vec{n}\times (\vec{A}\times \vec{n})$. A periodic boundary condition is applied for the side wall of the calculation geometry. At last, we carry out the meshing programme for discretizing the pre-defined 2D geometry, then numerically solve the transient wave equation coupled with the polarization field equation for obtaining the electric field of EOT via the inbuilt transient solver in the FR module of COMSOL Multiphysics. In this work, we will explore the spatial-temporal and transient-spectral properties of EOT using a two-slit plasmonic antenna milled in a Au film on top of silica substrate excited by the crossed femtosecond laser dual-beam.

## 3. Results and Discussion

**Figure 2:**
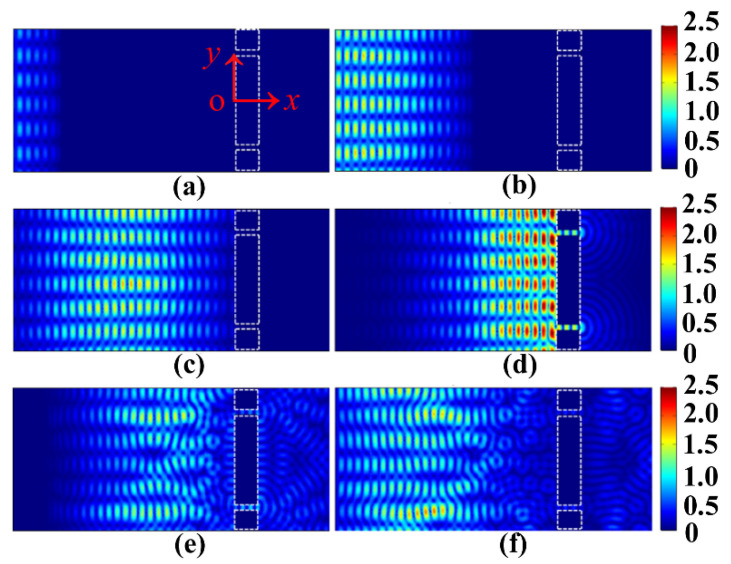
The 2D snapshots of the spatial electric field distribution of EOT through the two-slit plasmonic antenna excited by a crossed femtosecond laser dual-beam. The wavelength of the femtosecond laser is centered at 1.03 μm, the pulse duration is 20 fs, the phase difference Δφ is taken as π/3, and the angle of incidence of the laser is 20°. The distance between the two slits is 4 μm and the corresponding slit width is  0.25 μm. The Au film thickness is taken as 1 μm. Different evolution time (**a**) t=10 fs, (**b**) t=30 fs, (**c**) t=50 fs, (**d**) t=70 fs, (**e**) t=90 fs, (**f**) t=110 fs. All snapshots use the same spatial coordinates as in (**a**).

The 2D snapshots of the spatial electric field distribution of EOT through the two-slit plasmonic antenna excited by a crossed femtosecond laser dual-beam are shown in Figure 2. The electric field of the incident laser beam is normalized to one here. We can see that the propagating interference modes typically appear at an earlier evolution time of 10 fs (Figure 2a). Over time, the sustaining interference modes propagate continuously in space, which transmitted over a distance more than half of the incident free space at an evolution time of 30 fs (Figure 2b). With an evolution time of 50 fs, the propagating interference modes reach the front surface of the two-slit plasmonic antenna (Figure 2c). The surface plasmon cavity modes localized within the two slits typically appear at 70 fs (Figure 2d). What is more interesting is that we can see that the EOT is clearly transmitted into the silica substrate at this time. In fact, the two-slit surface plasmon resonance plays an important role in the formation of the cavity modes in the horizontal slit of the two-slit antenna, which in turn supports the generation of EOT that is propagated into the silica substrate. With an evolution time of 90 fs, the EOT propagates into space entirely in the silica substrate, while simultaneously accompanying the perturbation by reflecting waves from the front surface of the two-slit plasmonic antenna (Figure 2e). The dephasing perturbations become more and more prominent, eventually forming random patterns in the proximity of the front surface of Au film slits at an evolution time of 110 fs (Figure 2f). It should be noted that the phase difference for the crossed femtosecond laser dual-beam is taken as π/3 here. In fact, however, a controlled phase difference can have more diverse consequences for the EOT of the plasmonic two-slit antenna. In the following section, we focus on exploring the ultrafast dynamics of EOT by controlling the phase correlation of the crossed femtosecond laser dual-beam to explore the transient EOT with symmetry-tunable capabilities.

**Figure 3:**
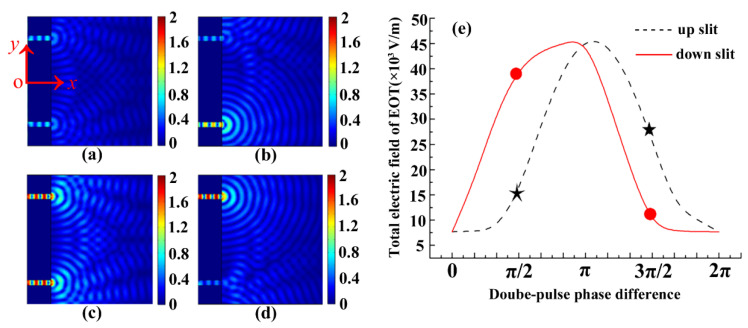
The 2D snapshots of the spatial electric field distribution of transient EOT with symmetry-tunable properties for the two-slit plasmonic antenna with respect to the phase difference of a crossed femtosecond laser dual-beam. The wavelength of femtosecond laser is centered at 1.03 μm, the pulse duration is 20 fs, and the angle of incidence of the laser is taken to be 20°. The two-slit is spaced at  4 μm, and corresponding slit width is  0.25 μm. The Au film thickness is taken to be 1 μm. (**a**) ∆φ=0, 2π; (**b**) ∆φ=π/2; (**c**) ∆φ=π; (**d**) ∆φ=3π/2. All snapshots use the same spatial coordinates as in (**a**). (**e**) EOT as a function of the phase difference of the crossed femtosecond laser dual-beam.

The 2D snapshots of the spatial electric field distribution of transient EOT with symmetry-tunable properties for the two-slit plasmonic antenna with respect to the phase difference of the crossed femtosecond laser dual-beam are shown in Figure 3. Here, the EOT images are extracted with a time of 82 fs, which corresponds to the maxima of the transient EOT electric field through the plasmonic two-slit antenna. The respective angle of incidence for the crossed femtosecond dual-beam are identically taken as 20°. At the phase differences of 0 and 2π, the electric field of EOT is symmetrically distributed at the respective slits of the two-slit plasmonic antenna (Figure 3a). Nevertheless, the symmetry of the EOT electric field is completely broken through the two slits at the phase difference of π/2 (Figure 3b). More interestingly, the symmetry of the EOT of the two-slit plasmonic antenna is restored at a phase difference of π (Figure 3c). We can see that the asymmetrically enhanced EOT evidently appears at the up-slit (Figure 3d) at a dual-beam phase difference of 3π/2. This can be further illustrated using the EOT as a function of the phase difference of the crossed femtosecond laser dual-beam as shown in Figure 3e. Here, the EOTs are defined by spatial integration of the electric field over the respective slit regions at the exits of the two-slit plasmonic antenna, and the incident electric field is taken as 3 × 10^4^ V/m. We can see that the EOT curves of the up- and down-slit overlap at a phase difference of 0, π, and 2π, indicating that the symmetrical properties of EOT for the two-slit plasmonic antenna can be well assured at the optimized conditions of phase correlation. However, the symmetry of the EOT can clearly be affected and broken if the phase difference is controlled at π/2 or 3π/2, respectively. This can be explained as tunable interference dynamics by phase control of the pluralistic coupling of surface plasmon polariton waves with the crossed dual-beam. For phase differences of 0, π,  and  2π, the dynamic interference between the surface plasmon waves with the crossed dual-beam can be well constructed in the slits of a two-slit plasmonic antenna, leading to the observed symmetry of EOT. However, once the phase difference for constructive interference deviates, especially at π/2 or 3π/2, the surface plasmon polariton waves can be asymmetrically excited with the crossed dual-beam in slit regions, leading to enhanced asymmetry of the spatial propagation of EOT. In fact, the results of two slits irradiated by dual-beam results cannot be simply realized by using a single slit irradiated by a single beam. In this work, we concentrated the topic for a better understanding of the dynamics of EOT from two-slit plasmonic antenna.

**Figure 4:**
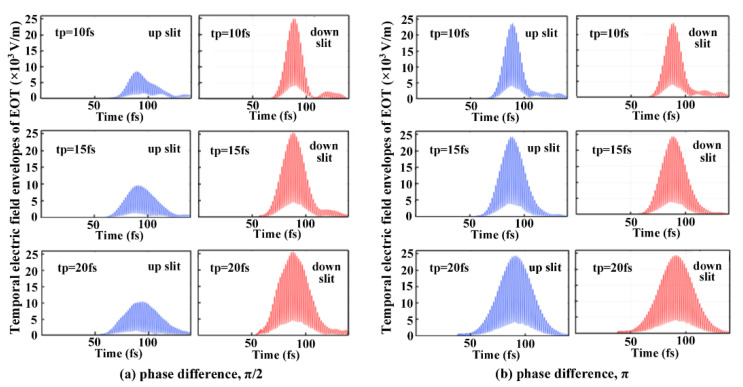
The electric field envelopes of EOT through the respective slits of the two-slit plasmonic antenna excited by the crossed femtosecond laser dual-beam with different pulse duration (**a**) phase difference, π/2; (**b**) phase difference, π. The femtosecond laser wavelength is centered at 1.03 μm, and the laser incident angle is taken as 60°. The two-slit is spaced at  4 μm, and corresponding slit width is  0.25 μm. The Au film thickness is taken as 1 μm.

The electric field envelopes of EOT through the respective slits of the two-slit plasmonic antenna excited by the crossed femtosecond laser dual-beam with respect to different pulse duration are shown in Figure 4. Here, the pulse envelopes from the up- and down-slits of the two-slit plasmonic antenna are obtained via spatially integrating the electric field over the respective slit area at the exits of the two-slit plasmonic antenna. The incident electric field is taken as a value of 3 × 10^4^ V/m. We can see that at a phase difference of π/2, the asymmetric temporal envelope of the EOT can arise from up- and down-slits at pulse durations of 10 fs, 15 fs, and 20 fs, respectively (Figure 4a). Nevertheless, symmetrical EOT typically occurs for pulse durations of 10 fs, 15 fs, and 20 fs at a pulse phase difference of π (Figure 4b). We can further see that the EOT electric field envelopes clearly deviate from the Gaussian profile for the phase differences of π/2 and π, especially at the tail of a femtosecond pulse. In particular, the distortion of the electric field envelope becomes even more severe at the shorter 10 fs pulse duration compared to the longer 15 fs and 20 fs pulse durations. It originates from the dynamic interference that strengthens the temporal reshaping of EOT by the two-slit plasmonic antenna, potentially leading to the observed tail distortion of the EOT electric field envelope. The results can be fundamentally helpful for understanding and controlling the transient EOT for exploring advanced applications in the fields of dynamic SERS bio-sensors, ultrafast light switches, and dynamic imaging, etc.

**Figure 5:**
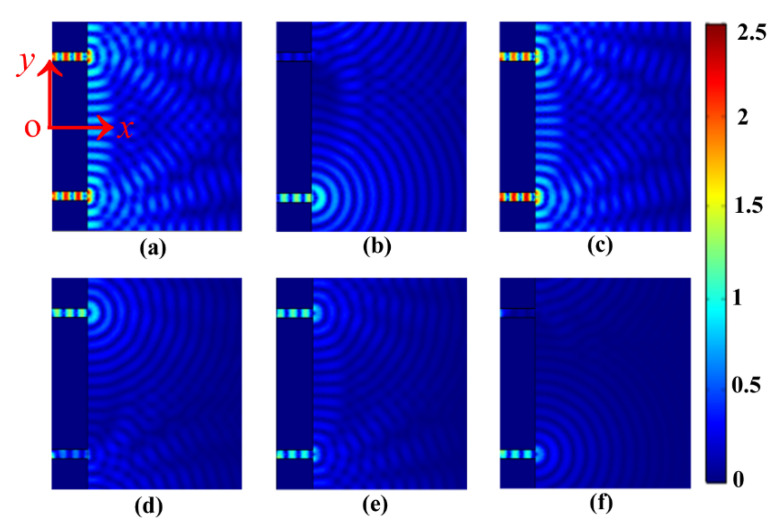
The 2D snapshots of the spatial electric field distribution of transient EOT through the two-slit plasmonic antenna with respect to the incident angle of the crossed femtosecond laser dual-beam. The femtosecond laser wavelength  1.03 μm, the pulse duration  20 fs. The two slits are spaced at  4 μm, and respective slit width  0.25 μm, the Au film thickness 1 μm. (**a**)  θ=0°; (**b**)  θ=20°; (**c**)  θ=30°; (**d**) $\theta =40{}^{\circ};\left(e\right)\;\theta =50{}^{\circ}$; (**f**) θ=60°. All snapshots use the same spatial coordinates as in (**a**).

The 2D snapshots of the spatial electric field distribution of transient EOT through the two-slit plasmonic antenna with respect to the incident angle of the crossed femtosecond laser dual-beam are shown in Figure 5. Here, the electric field images are extracted from the EOT at a time of 82 fs. The electric field of the incident laser beam is normalized to one here. The phase difference is set to zero to study the basic properties of the transient EOT in relation to the oblique angle of incidence of the crossed dual-beam. It can be seen from Figure 5a,c,e that the electric field penetrates equally into the respective slits, enabling the EOT with equal energy distribution by the two-slit plasmonic antenna at incidence angles of 0°, 30°, and 50°, respectively. Moreover, the transient electric field of the EOT is particularly enhanced at the angles of incidence of 0° and 30°. More importantly, it can be seen that the incident angle is fixed at 20°, 40°, and 60°, and the transient electric field of EOT exhibits asymmetric distribution through the respective slits of the two-slit plasmonic antenna. The electric field from the down-slit is clearly stronger than that from the up-slit at 20° and 60°, as can be seen in Figure 5b,f. In contrast, the electric field of EOT at the down-slit becomes weaker than at the up-slit at 40°, as seen in Figure 5d. This is explained as tunable interference dynamics between the surface plasmon polariton waves and the incident dual-beam, allowing dynamic modulation of the EOT with optimized oblique incidence of the dual-beam. The constructive phase matching for the interference of the two-slit plasmonic antenna can be well supported at the optimized oblique incidence angle of 30° for the current geometry of the simulations. However, phase detuning can be developed for destroying the interference dynamics when the incident angle deviates from the specific angle, leading to the degeneration of EOT intensity, which can be particularly detuned at the asymmetric modes at an angle of incidence of 20°, 40°, or 60°. The results may provide a basis for understanding the transient EOT characteristics by modifying the laser incident angle under the crossed femtosecond laser dual-beam excitation. It should be noticed that the EOT for a single slit with a single laser beam exhibits a monotonous decrease in the electric field of EOT, increasing the incident angle from 0° to 60°, not shown here.

**Figure 6:**
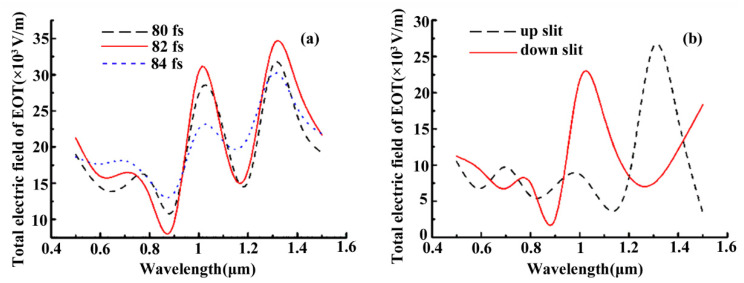
(**a**) The transient electric field of the EOT spectra of the two-slit plasmonic antenna at different computation time. (**b**) The transient electric field of the EOT spectra extracted from up-slit and down-slit at a calculated time of t = 82 fs. The two-slit spacing d=4 μm, and respective slit width  w=0.25 μm, Au film thickness 1 μm.

The transient spectra of EOT through the two-slit plasmonic antenna with respect to the incident angle of the crossed femtosecond laser dual-beam are shown in Figure 6. Here, the spectra are obtained by spatial integration of the electric field over the respective slits area at the exits of the two-slit plasmonic antenna, written by $E_{total}=\frac{1}{w}\int_{slit1}^{}{\left|E\left(y,t\right)\right|}_{EOT}\cdot dy+\frac{1}{w}\int_{slit2}^{}{\left|E\left(y,t\right)\right|}_{EOT}\cdot dy$. The incident electric field is taken as a value of 2 × 10^4^ V/m. It can be seen from Figure 6a that the transient spectra for EOT exhibit a two-peak profile in the near-infrared regime. The spectrum reaches a value corresponding peak at a wavelength of 1.32 μm at 82 fs. However, the transient spectra of the EOT at wavelengths of 1.03 μm and 1.32 μm are clearly attenuated at both early and later times of 80 fs and 84 fs. Here, the phase difference of the crossed femtosecond laser dual-beam is given as π/2, which leads to the formation of the symmetry-broken EOT through the two-slit plasmonic antenna. We can see from Figure 6b that transient spectra can be quite inconsistent for the up- and down-slits of the two-slit plasmonic antenna. The transient EOT spectra extracted from the down-slit at the 1.03 μm laser wavelength are much larger than those from the up-slit of the two-slit plasmonic antenna. However, the reverse is true in that the spectral amplitude at the down-slit at a laser wavelength of 1.32 μm is much smaller than at the up-slit. It indicates that the transient two-peak spectra of the EOT from the two-slit plasmonic antenna can be flexibly switched on and off by tuning the laser wavelength of the femtosecond crossed dual-beam laser. The results can provide the basis for exploring the ultrafast spectral dynamics of the EOTs in the two-slit antenna, especially the symmetry-tunable characteristics of the EOTs through the two-slit plasmonic antennas for possible applications in the fields of time-resolution imaging, ultrafast plasmonic switch, and the transient enhancement of non-linear effects, etc.

## 4. Conclusions

We have theoretically investigated the ultrafast dynamics of EOT spatial-temporal dynamics using a two-slit plasmonic antenna made of a Au film coated on a silica substrate. It is demonstrated that the modified phase relevance with respect to the optimized incidence of the crossed femtosecond laser dual-beam can definitely be qualified for the manipulation of the spatial-temporal symmetry of EOT. This is attributed to the tunable dynamics by phase control of surface plasmon polariton wave interference with the incident dual-beam, allowing the dynamic modulation of EOT via femtosecond laser dual-beam irradiation. Moreover, it is revealed that the two-peak spectra of the transient EOT from the two-slit plasmonic antenna can be flexibly switched on and off by tuning the laser wavelength of the crossed femtosecond laser dual-beam. This study provides the foundation for exploring the dynamics of transient EOT for an extensive range of applications such as ultra-high time-resolution imaging, ultrafast light switch, and the transient amplification of non-linear effects, etc.

## Data Availability

Not applicable.
